# Complete genome sequences of *Brachyspira hyodysenteriae* strains B204 and JR80

**DOI:** 10.1128/mra.00168-25

**Published:** 2025-04-17

**Authors:** Judith Rohde, Michael Jarek, Ralph Goethe

**Affiliations:** 1 Department of Infectious Diseases, Institute for Microbiology, University of Veterinary Medicine Hannover 26556 , Hannover, Germany; 2 Genome Analytics, Helmholtz Centre for Infection Research 28336, Braunschweig, Germany; Queens College Department of Biology, Queens, New York, USA

**Keywords:** *Brachyspira hyodysenteriae*, hemolysis

## Abstract

We report on the complete genome sequences of strongly hemolytic *Brachyspira hyodysenteriae* strain B204 and weakly hemolytic *Brachyspira hyodysenteriae* strain JR80 as an important contribution for studying the genetic basis for these different phenotypes. The basionym for both strains is *Treponema hyodysenteriae*, and the homotypic synonym is *Serpulina hyodysenteriae*.

## ANNOUNCEMENT

Strongly hemolytic *Brachyspira hyodysenteriae* strains are causative for severe swine dysentery, an economically important enteric disease in fattening pigs. Here, we provide the complete genome of the strongly hemolytic *Brachyspira hyodysenteriae* strain B204, a former ATCC reference strain. Many research groups have used this strain as a virulent prototype of this bacterial species. The strain was isolated in the 1970s at the Institute of Veterinary Microbiology and Preventive Microbiology of Iowa State University, Ames, USA, from a pig with swine dysentery (1) and acquired from T.B. Stanton and S.B. Humphrey, National Animal Disease Center, Agricultural Research Service, US Department of Agriculture, Ames, USA.

Furthermore, the first complete genome sequence of a weakly hemolytic isolate of this species, *Brachyspira hyodysenteriae* strain JR80, is provided. It was isolated in 2015 at the Institute for Microbiology, University of Veterinary Medicine, Hannover, Germany, from a pig with mild non-bloody diarrhea (2). The strain was identified as *Brachyspira hyodysenteriae* by *nox*-gene sequencing, *cpn*60 sequencing, and whole-genome sequencing (2, 3). Hemolytic activity was quantified in relation to the strongly hemolytic type strain of the species, strain B78^T^ (2).

For DNA extraction, bacteria were grown anaerobically at 40°C for 22 h in Trypticase Soy broth (Oxoid, Wesel, Germany) with yeast extract (Oxoid, Wesel, Germany) (4), and DNA was extracted using the Wizard HMW DNA Extraction Kit (Promega, Walldorf, Germany) following the protocol for gram-negative bacteria. DNA quality was checked using a Bioanalyzer (Agilent).

DNA libraries for Illumina sequencing were prepared using the NEBNext Ultra II FS DNA Library Prep Kit. Library for ONT system was prepared using Ligation Kit (SQK-LSK 109) without fragmentation and size selection. Sequencing of the respective library followed on MiSeq (2 × 300 bp) with a 330×/220× fold coverage per sample and on ONT MinION MK1B Flongle (FLG001) with 1G/680Mbp with a 52×/93× fold coverage per sample. Illumina read quality was assessed using FastQC v.0.12.0 (https://www.bioinformatics.babraham.ac.uk/projects/fastqc), while ONT reads were base called with Dorado v0.7.0 model dna_r9.4.1_e8_hac@v3.3 (https://github.com/nanoporetech/dorado) and underwent filtering with a minimum subread length of 1000 bp and quality via Filtlong v.0.2.1 (https://github.com/rrwick/Filtlong).

Hybrid assembly of both read types was performed using Unicycler 0.5.0 (5). The resulting contigs were also circularized by Unicycler, adjusted to start consistently at a designated gene, and represented the complete genome’s chromosome and plasmid for both strains. Genome annotation was conducted through the NCBI Prokaryotic Genome Annotation Pipeline (6), utilizing the best-placed reference protein set, GeneMarkS-2+. Unless noted otherwise, default parameters were applied for all software tools. Sequencing statistics and annotation details are shown in Table 1.

**TABLE 1:** Genome assembly statistics for *Brachyspira hyodysenteriae* strains B204 and JR80

Sequencing method or annotation	Data for *B. hyodysenteriae* strain	
	B204	JR80
ONT MinION sequencing	MK1B (FLG001)	MK1B (FLG001)
No. of subreads	36.812	16.047
Avg read length (bp)	5,146	9,628
Avg quality score	15% > Q20	18% > Q20
Sequencing throughput (bp)	157M	117M
Avg coverage depth (×)	52	39
SRA accession no.	SRR30838155	SRR30837070
MiSeq sequencing	MiSeq (2 × 300)	MiSeq (2 × 300)
No. of reads	3,327,930	2,261,570
Avg read length (bp)	301	301
Avg quality score	86% > Q30	87% > Q30
Sequencing throughput (bp)	1.0G	680M
Avg coverage depth (×)	330	220
SRA accession no.	SRR30838156	SRR30837071
PGAP annotation	2024-04-27.build7426	2024-07-18.build7555
chromosome	B204	JR80WH
GenBank accession no.	CP170528	CP171216
Length (bp)	3.004.220	3.041.621
No. of rRNAs	1,1,1 (5S, 16S, 23S)	1,1,1 (5S, 16S, 23S)
No. of tRNAs	34	34
No. of genes	2,662	2,708
No. of coding sequences (CDSs)	2,622	2,668
No. of protein coding genes	2,601	2,619
GC content (%)	27	27
plasmid	pBHB204	pBHJR80
GenBank accession no.	CP170529	CP171217
Genome length (bp)	35.914	32.585
No. of coding sequences	33	30
No. of protein coding genes	33	30
GC content (%)	27	27

## Data Availability

This whole-genome project at GenBank can be found under the accession numbers given in Table 1. The version described in this paper is the first version. In addition to the deposition of the sequencing data, we also deposited both strains at the German Cell Culture Collection DSMZ, Braunschweig, Germany, with the accession numbers DSM 118648 for strain B204 and DSM 118649 for strain JR80.
